# Evaluating the Prognostic Value of Islet Autoantibody Monitoring in Islet Transplant Recipients with Long-Standing Type 1 Diabetes Mellitus

**DOI:** 10.3390/jcm10122708

**Published:** 2021-06-19

**Authors:** Roi Anteby, Aaron Lucander, Piotr J. Bachul, Jordan Pyda, Damian Grybowski, Lindsay Basto, Gabriela S. Generette, Laurencia Perea, Karolina Golab, Ling-jia Wang, Martin Tibudan, Celeste Thomas, John Fung, Piotr Witkowski

**Affiliations:** 1 Department of Surgery, The University of Chicago, Chicago, IL 60637, USA; ranteby@hsph.harvard.edu (R.A.); aaron.carl.lucander@gmail.com (A.L.); Piotr.Bachul@uchospitals.edu (P.J.B.); damian.grybowsky@gmail.com (D.G.); lindsay.basto@uchospitals.edu (L.B.); Gabriela.Generette@uchospitals.edu (G.S.G.); lperea@surgery.bsd.uchicago.edu (L.P.); kgolab@surgery.bsd.uchicago.edu (K.G.); lwang6@surgery.bsd.uchicago.edu (L.-j.W.); mtibudan@surgery.bsd.uchicago.edu (M.T.); jfung@surgery.bsd.uchicago.edu (J.F.); 2 Faculty of Medicine, Tel Aviv University, Tel Aviv 6997801, Israel; 3 Harvard School of Public Health, Boston, MA 02115, USA; 4 Department of Surgery, University of Alabama at Birmingham, Birmingham, AL 35233, USA; 5 Department of Surgery, Beth Israel Deaconess Medical Center, Boston, MA 02215, USA; jordanpyda@gmail.com; 6 Department of Medicine, The University of Chicago, Chicago, IL 60637, USA; cthomas5@bsd.uchicago.edu

**Keywords:** type 1 diabetes mellitus, allogenic islet cell transplantation, autoantibodies, GADA, pancreas

## Abstract

(1) Background: The correlation between titers of islet autoantibodies (IAbs) and the loss of transplanted islets remains controversial. We sought to evaluate the prognostic utility of monitoring IAbs in diabetic patients after islet transplantation (ITx); (2) Methods: Twelve patients with Type 1 diabetes mellitus and severe hypoglycemia underwent ITx. Serum concentration of glutamic acid decarboxylase (GAD), insulinoma antigen 2 (IA-2), and zinc transport 8 (ZnT8) autoantibodies was assessed before ITx and 0, 7, and 75 days and every 3 months post-operatively; (3) Results: IA-2A (IA-2 antibody) and ZnT8A (ZnT8 antibody) levels were not detectable before or after ITx in all patients (median follow-up of 53 months (range 24–61)). Prior to ITx, GAD antibody (GADA) was undetectable in 67% (8/12) of patients. Of those, 75% (6/8) converted to GADA+ after ITx. In 67% (4/6) of patients with GADA+ seroconversion, GADA level peaked within 3 months after ITx and subsequently declined. All patients with GADA+ seroconversion maintained long-term partial or complete islet function (insulin independence) after 1 or 2 ITx. There was no correlation between the presence of IAb-associated HLA haplotypes and the presence of IAbs before or after ITx; (4) Conclusions: There is no association between serum GADA trends and ITx outcomes. IA-2A and ZnT8A were not detectable in any of our patients before or after ITx.

## 1. Introduction

Type 1 diabetes mellitus (T1D) is a widespread economically and socially significant autoimmune disorder [1]. The incidence of T1D is rapidly increasing, with 3–5% more cases each year, and has doubled over the last two decades and thus the development of more effective and reliable therapies is essential for not only optimizing patient care but to effectively address the global disease burden [2].

Although T1D results from the T lymphocyte-mediated destruction of insulin-producing β-cells in the pancreatic islets of Langerhans, B lymphocytes and autoantibodies also play a role in islet autoimmunity [3]. Clinical symptoms are preceded by the appearance of autoantibodies targeting islet-related antigens (IAbs). The presence of two or more of the four major islet autoantibodies (IAbs), targeting insulin (IAA), glutamic acid decarboxylase 65 (GADA), insulinoma antigen 2 (IA-2A), and zinc transport 8 (ZnT8A), is now sufficient to diagnosis presymptomatic stage of T1D and confers a 70% risk of developing clinical T1D within 10 years and a nearly 100% probability of disease manifestation within two decades [2,4]. Seroconversion to autoantibody positivity may mark the onset of islet autoimmunity and occurs heterogeneously among age groups. While young children are likely to develop IAA first, adolescents and young adults frequently present with GADA, and individuals with certain genetic backgrounds are particularly susceptible [2]. GADA seropositivity has been associated with the HLA DR3-DQ2 haplotype, and IAA and IA-2A expression with the HLA DR4-DQ8 haplotype [5].

Islet and pancreas transplantation are currently the only curative treatments for T1D, but their ability to restore long term insulin-independence is hindered by both allogenic rejection and recurrent islet autoimmunity, alone or in combination. Autoimmune T1D Recurrence (T1DR) is an underdiagnosed cause of pancreas or islet graft loss [6]. In pancreas transplantation, two cardinal features can assist in diagnosing T1DR: (1) selective loss of insulin secretion, with unchanged exocrine function; (2) confirmatory biopsy demonstrating focal insulitis and/or β-cell loss [7]. However, in islet allotransplantation (ITx) the former is inapplicable, and the latter is practically not impossible to obtain [8].

Due to the well-defined significance and prognostic role of IAbs in T1D, there is growing interest in their use as possible early detection markers for T1DR after pancreas or islet transplantation [9]. Previous studies identified an association between autoantibodies and long-term islet function, with the upregulation or reappearance of IAbs linked to lower graft survival [10,11,12] or decline in glycemic control [6]. This correlation has been reported in both pancreatic graft transplantation and ITx [8,13,14]. However, other studies did not find an association between post-transplant autoantibody dynamics and clinical outcomes, thus challenging the usefulness of IAbs as effective biomarkers for T1DR [15,16,17].

In this study, we sought to interpret the prognostic value of IAbs for predicting graft failure in our cohort of islet transplant recipients with T1D. Given the integral role of neutrophils and T cells in disease pathogenesis, we also evaluated the effect of inhibiting leukocyte and lymphocyte trafficking via the CXCR1 and CXCR2 inhibitor Reparixin on islet graft failure and insulin-independence. The instant blood-mediated inflammatory reaction (IBMIR) is a major cause of tissue loss following islet administration, and early prevention of neutrophil and lymphocyte trafficking to the site of transplantation could support the survival and engraftment of a greater percentage of islets [18]. Given the importance of identifying markers of graft failure and the potential value of Reparixin in islet transplantation, we additionally sought to determine whether Reparixin treatment could affect the expression of IAbs.

## 2. Materials and Methods

### 2.1. Islet Transplant Patients and Baseline Characteristics

Twelve patients with long-standing brittle T1D experiencing severe hypoglycemic episodes received ITx between 2013 and 2015 at the University of Chicago Medical Center. Patients were randomly stratified into two treatment groups in a double-blind controlled trial as previously described [19]. Eight patients received the CXCR1/2 inhibitor Reparixin, while the other 4 patients were given placebo in addition to standard immunosuppression. Reparixin (2.772 mg/kg patient body weight) (Dompe, Italy) or a placebo (0.9% NaCl) was continuously infused intravenously via central line, starting at least 6 (up to 18) hours prior to ITx and continuously over 7 days (168 h). Immunosuppression for all cases involved anti-thymocyte globulin (ATG) for induction and tacrolimus with mycophenolate mofetil for maintenance. ATG (6 mg/kg) was delivered in divided doses intravenously immediately prior to transplantation and for up to 7 days following the procedure. Islets were isolated from brain-dead donors and transplanted after confirming negative virtual and CDC standard cross match and blood group compatibility [19]. Islet were infused intraportally via a percutaneous catheter placed by interventional radiology (see Supplementary Materials for a detailed description of the transplantation procedures).

### 2.2. Islet Graft Function Outcome Measures and Definitions

Detection of fasting C-peptide above 0.1 ng/mL was used to confirm the presence of the islet cell graft function: partial islet graft function, when patient still required some exogenous insulin supplementation, and full islet graft function, when patient was insulin independent. A fasting C-peptide level of 0.1 ng/mL was considered as a threshold to define islet cell graft function. Insulin independence was defined as optimal glucose control without the need for insulin supplementation leading to hemoglobin A1c (HbA1c) < 6.5, postprandial blood glucose < 180 mg/dl, and fasting blood glucose < 140 mg/dl. Stable islet graft function was determined if the patient remained insulin independent or daily insulin requirements remained unchanged. Declining islet graft function or islet graft dysfunction were determined based on re-initiation of the insulin supplementation in insulin independent patients or based on increasing insulin requirements in patients with partial islet graft function. Islet graft loss or lack of the islet graft function were determined when serum C-peptide was undetectable. C-peptide was measured by chemiluminescent immunoassay on an Immulite 2000 (Siemens Healthcare Diagnostics Products). The assay measures serum with as little as 0.033 pmol/mL and interassay CV of 3%, intra of 2%.

### 2.3. IAbs and HLA Antigen Measurements

Expression levels of IAbs GADA, IA-2A, and ZnT8A were assessed prior to ITx, 0, 7, and 75 days and every 3 months post-operatively. We collected a total of 256 serum samples, with a median of 20 (range 6–34) samples per patient. The correlation between IAbs level and islet graft function was assessed retrospectively. HLA antigens were established in the American Society of Histocompatibility and Immunogenetics at the University of Chicago. HLA low resolution was performed by reverse sequence specific oligonucleotide probe hybridization (rSSOP)-based microarray assay, while HLA high resolution typing was performed by sequence-based typing (SBT).

### 2.4. Statistical Analyses

Descriptive statistics included: median, range, and percentages. Normality was assessed using the Shapiro–Wilk test. Student’s *t*-test was used to compare continuous parametric variables, Mann–Whitney U test was used to compare non-parametric continuous variables, and Fisher’s exact test was used to compare categorical variables. A conventional *p*-value ≤ 0.05 was considered to be statistically significant. All analyses were performed using Statistica software (v 12.0, StatSoft, CA, USA).

## 3. Results

Median age was 43 years (28–56) and median duration of T1DM at time of treatment was 31.5 years (15–47). Patient characteristics in the Reparixin and placebo group were similar (Table 1). The median follow-up time was 56 months (24–66). Four patients received one ITx and eight patients received two ITx. The median follow-up time was 56 months (24–66). At last follow-up, 33% of the patients (*n* = 4) had functioning islet grafts (i.e., responders), and the rest (67%, *n* = 8) were considered non responders at last follow-up visit. Of these, four (50%) had de novo DSA/AMR and 50% had unexplained islet function loss.

IA-2A and ZnT8A not detected pre- or post-IT. Autoantibodies targeting IA-2 and ZnT8 were not detected in the peripheral blood of all 12 islet transplant patients at all assessed time points pre- and post-transplantation.

### 3.1. Transient GADA Increase Following ITx

**Figure 1:**
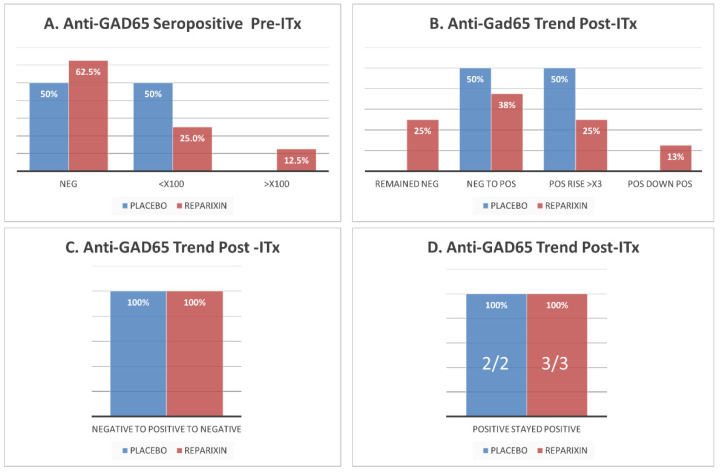
GADA seropositivity of islet transplant (IT) recipients pre- and post-IT in Reparixin vs. placebo group. (**A**) GADA seropositivity of islet recipients prior to IT. Most of the patients were GADA negative prior to IT in both groups. (**B**) Trend of GADA expression after IT in relation to pre-IT. Most patients developed or increased their titers of GADA in both groups. Persistence of GADA after IT: All patients who were GADA negative pre-IT, developed GADA after IT, but cleared them within 6 months. (**C**). All patients who were GADA positive pre-IT remained seropositive after IT (**D**). ITx, islet transplantation.

Most patients (8/12, 66%) were GADA negative prior to islet transplantation and most of them (6/8, 66%) converted to seropositivity or increased antibody titers following the procedure (Figure 1A,B). Among the patients that converted to GADA seropositive by day 7 after ITx, most converted back to seronegative by 1 year after transplantation and maintained long-term (>4 years) stable partial or complete islet function (Figure 1C). One patient who developed antibody-mediated rejection by day 7 became GADA seropositive at the same time, with an extremely high titer, and remained persistently seropositive with low autoantibody titers. After his second transplant, he remained insulin-free for 9 months, but his GADA titers remained stably low and persisted without elevation even when he gradually lost his islet graft following an acute cytomegalovirus infection. All patients expressing GADA prior to ITx remained seropositive for the duration of our study and did not become seronegative (Figure 1D).

### 3.2. Reparixin Does Not Significantly Alter GADA Expression Dynamics Post-ITx

Prior to ITx, GADA levels did not vary between patients receiving Reparixin or placebo, and most patients were GADA negative (65% and 50%, respectively) (Figure 1A). Additionally, no difference was observed for trends of GADA levels after ITx between these groups. Most patients in both groups converted from GADA seronegative to seropositive or increased antibody titers (5/8 (62.5%) and 4/4 (100%), respectively) (Figure 1B). In both groups, most patients that converted to GADA seropositive by day 7 after ITx (2/3 (66%) and 2/2 (100%), respectively) also converted back to seronegative post-transplant by 1 year and maintained long-term (>4 years) stable partial or full islet function (Figure 1C). All patients from both groups expressing GADA prior to IT remained seropositive for the duration of our study and did not convert to seronegative (Figure 1D).

### 3.3. GADA Expression Post-ITx Does Not Predict Insulin Independence or Graft Failure

**Figure 2:**
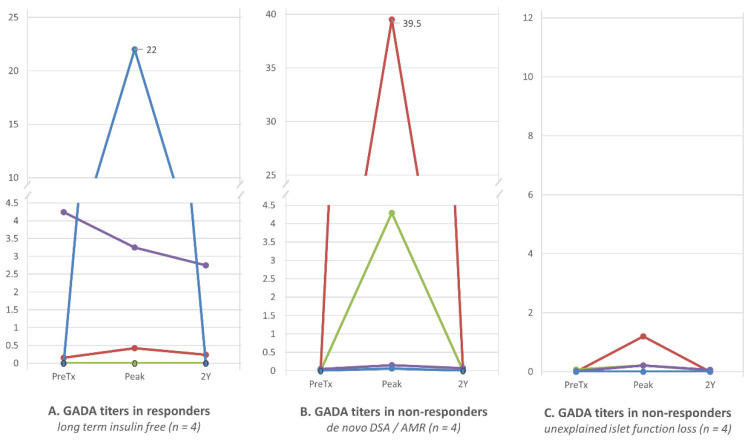
Trends of GADA in the islet transplant (ITx) responder and non-responder groups. There was no clear pattern of GADA level trends in relation to stability (responders) or declining or lack of islet graft function (non-responders) of islet function. Variability in GADA levels was similar in each group regardless of islet function and mechanism of islet injury. (**A**): GADA titers in responders. Four (4) patients who presented stable long-term (>2 years) insulin independence after only one IT (*n* = 4). Each patient displayed a completely different trend of GADA. One patient who was seronegative remained so long-term. Despite stable islet function allowing for insulin independence over 5 years, another patient spiked GADA to 22 nmol/L at day 14 after IT and remained seropositive for 1 year. The remaining two patients were seropositive prior to IT and remained so afterwards, one with low and the other with much higher levels (4.24–2.68 nmol/L). (**B**): GADA titers in non-responders, who developed de novo DSA and loss of islet function (*n* = 4). All patients in this group developed GADA with rising levels in parallel to DSA; however, GADA trends in this group were very similar to trends in responders without any DSA and islet dysfunction. (**C**): GADA titers in non-responders, who gradually loss their islet function without obvious reason (*n* = 4). One patient remained GADA seronegative while losing partial islet graft function after each of three subsequent transplants. The remaining 3 patients presented either with persistent elevation or without an elevation of GADA following IT. GADA trends between patients in this group varied, and variability was similar to that observed in other groups. Autoantibody levels measured in nmol/L.

No difference was observed for trends of GADA titers between 4 patients with stable long-term insulin independence after ITx (responders) and the remaining 8 who experienced a decline in islet graft function (non-responders). Among responders, one patient was GADA seronegative prior to ITx. Immediately post-IT he had a spike in GADA to 22 nmol/L (normal 0–0.02 nmol/L), and thereafter gradually returned to permanent seronegativity (Figure 1A). A second patient expressed high GADA levels prior to ITx (4 nmol/L) and maintained a high titer post-ITx. The third responder expressed low levels of GADA prior to ITx and experienced a six-fold titer increase post-ITx which gradually stabilized at 2–3 times the normal level. The fourth responder was GADA seronegative prior to ITx and has remained seronegative for over 4 years. Trends of GADA titers among non-responders were as variable as those among responders (Figure 2B). The development of antibody-mediated rejection or donor-specific antibody did not correlate with the expression of GADA following ITx (Figure 2C) in our cohort.

### 3.4. No Correlation between IAb-Associated HLA Haplotypes and Trends of IAbs after Islet Transplantation

GADA associated HLA DR3-DQ2 haplotype was not present in any of our 12 patients. All but one patient in each group presented with GADA prior to ITx or developed the autoantibody afterwards. In contrast, HLA DR4-DQ8 haplotype associated with IA-2A was present in most of the patients (6/8 (75%) and 1/4 (25%) patients in the Reparixin and placebo groups, respectively).

## 4. Discussion

Although T1D is commonly managed via exogenous insulin administration, some patients are unable to maintain appropriate glycemic control and require islet engraftment to avoid long-term diabetes-related complications. Our series of 12 patients suffered from frequent severe hypoglycemic episodes and consequently received islet allografts to promote insulin independence. Following only one islet transplantation, four patients achieved stable long-term insulin independence without a decline in islet graft function. Eight patients experienced partial graft function or graft failure and required additional islet transplantations.

The prognostic role of autoantibodies in the setting of islet and pancreas transplantation is controversial. In our series, most patients were GADA seronegative prior to transplantation and became seropositive or had increased antibody titers within seven days of the procedure, but returned to seronegativity within one year, maintaining stable graft function. In contrast, islet transplant recipients expressing GADA prior to transplantation remained seropositive after transplantation for the duration of this study. Autoantibodies targeting IA-2 and ZnT8 were not detectable in the peripheral blood of islet transplant recipients prior to or following transplantation. In our current analysis, insulin-independence did not appear to be associated with GADA titers following transplantation. Reparixin therapy did not improve the metabolic outcomes of islet grafts in a multicenter trial; however, this treatment modality may still have some beneficial effect, as observed in our cohort [19,20]. Nevertheless, Reparixin did not appear to affect trends of GADA titers pre- and post-IT in our patients.

Consistent with prior findings, antibodies targeting GAD were found to be more prevalent than those targeting IA-2 or ZnT8 [14]. While GAD is highly expressed at the protein level by islet cells, IA-2 protein expression is more restricted and ZnT8 exists mainly as mRNA. This corresponds to the increased frequency of GADA seropositivity among T1D patients, as GAD protein epitopes are more available for T and B cell targeting than those of the other antigens [3]. Notably, this may correspond to the induction of GADA following transplantation observed in our study. Trauma associated with islet reperfusion injury during the islet engraftment may re-expose GAD-specific memory B cells or long-lived plasma cells to associated antigens, initiating autoantibody production, while IA-2 and ZnT8 levels are insufficient to stimulate naive or memory cell responses. Additionally, trauma may induce the secretion of danger signals such as HMGB1 and pro-inflammatory cytokines that promote the re-activation of pathogenic lymphocytes [21].

It is still not clear whether IAbs are direct mediators of islet destruction or simply passive observers induced secondary to antigen exposure to the immune system during islet cell damage [14]. IAbs theoretically have the potential to be utilized for the diagnosis and monitoring of recurrent autoimmunity, which prompted our current investigation.

While GADA re-appeared or increased following transplantation in most patients from our cohort, we did not observe a correlation between GADA levels and islet allograft failure. Previous studies on autoantibodies and their role as markers for islet cell graft outcome have demonstrated mixed results. Piemonti et al. analyzed a cohort of 59 T1D islet transplant recipients, demonstrating an association between posttransplant autoantibody increases and significantly lower graft survival (hazard ratio of 5.21) [8]. In their study, an increase in any of the measured autoantibodies (GADA, ZnT8A, or IA-2A) was also predictive of a shorter duration of insulin independence [8]. Notably, pre-transplant autoantibody status did not influence the functional outcome of islet transplantation. A similar trend was shown by Bosi et al., who reviewed a series of 36 T1D recipients of islet allografts and found that GADA reappeared first but IA-2A and ZnT8A were the strongest predictors of graft failure [13]. Therefore, IA-2A and ZnT8A may be more specific surrogate markers for recurrent autoimmunity than GADA [5]. Since GADA did not correlate with islet graft function and we did not observe any detectable IA-2A and ZnT8A in our patients at any timepoint, it is possible that none of the patients in our cohort experienced recurrent autoimmunity.

Overall, the correlation between IAbs and outcomes of pancreas and islet transplantation remains controversial. In two European retrospective reviews of a total of 51 T1D patients that received ITx, investigators did not find a correlation between autoantigen reactivity after ITx and graft function in the first 6 [16] and 12 months following implantation [17]. Similarly, no trends were noticed in a smaller series published by Roep et al. [15]. Additionally, a large study of 135 T1D pancreas graft recipients, with a long-term follow-up (median = 6 years), failed to show a connection between autoantibodies dynamics and graft survival. However, it did show worse outcomes, in the form of higher hemoglobin A1C and lower C-peptide levels, in patients with new-onset or rising levels of autoantibodies after transplantation [6]. In contrast, a more recent study was able to detect reduced pancreas graft survival in patients with de-novo GADA compared to those without [14].

Observed differences in islet allograft outcomes may be attributed to systematic variation between studies, including differences in patient cohort characteristics, follow-up time, islet allograft composition, immunosuppression regimens, and surgical procedures.

Although a multicenter study did not show a positive effect of Reparixin on islet engraftment, under special conditions such an effect might be observed. Reparixin inhibits the alpha and beta subunits of the interleukin-8 (IL-8) receptor, antagonizing IL-8-associated programming or chemotaxis by pathogenic neutrophils and T cells. Since these processes are more important for direct neutrophil and cytotoxic T lymphocyte-mediated destruction of islets than TH2-mediated B cell help in germinal centers, this inhibitor may support islet survival without affecting autoantibody responses. The value of Reparixin in the prevention of the instant blood-mediated inflammatory response (IBMIR) must be considered and remains to be demonstrated. In our study, the statistical significance of our results is limited by a low sample size. Reparixin treatment might have affected long-term islet function in our cohort, but not GADA seropositivity comparing to placebo (Cell Tx).

The small number of patients and the use of a single immunosuppression protocol are clear limitations of our study. It is possible that patients with certain genetic characteristics, or treated via alternative immunosuppression regimens, would show a correlation between IAbs and islet graft function. Furthermore, although our follow-up was considerably long (median of 4 years), we acknowledge that longer follow-up periods might reveal a possible association.

Since T cell-mediated islet destruction is a proven element of recurrent autoimmunity, future studies should involve the analysis of islet autoantigen-specific T cell clones in the peripheral blood in conjunction with the assessment of autoantibody levels and clinical markers of disease progression. Notably, patients without islet-antigen-autoreactive cytotoxic CD4+ T cells prior to transplantation are much less likely to achieve long-term insulin-independence [9]. Since autoreactive T cell clones have been demonstrated to expand during homeostatic proliferation following induction, the concomitant assessment of T cell and autoantibody levels may further elaborate the mechanistic involvement of GADA, IA-2A, and ZnT8A in disease pathogenesis. Given the ability of certain immunosuppressive regimens to affect the development of auto- and allo-reactivity, exploring the dynamics of T and B cell responses to islet transplantation in the context of different pharmacologic regimens (Reparixin versus placebo versus anti-TNF agents; anti-thymocyte globulin versus basiliximab; rapamycin versus tacrolimus) could contribute to characterizing the effects of specific drugs on the immune system and their safety and efficacy for the prevention of islet graft failure [22,23]. We anticipate that the identification of reliable markers of islet graft failure will depend upon the broad and combined characterization of T and B cell phenotypes, and that these data could lead to improved therapeutic approaches.

## 5. Conclusions

In our single center 12-patient cohort, GADA levels were not associated with pancreatic islet transplant outcomes during a median 4 year follow-up. Levels of islet autoantibodies targeting IA-2 and ZnT8 were non-detectable in all patients prior to and after transplantation and also did not correlate with transplant outcome. Further multi-institutional research with larger patient cohorts, different immunosuppressive regimens, and longer follow-up are needed to test the generalizability of our findings.

## Figures and Tables

**Table 1:** Characteristics of islet transplant recipients. Demographic characteristics and disease course of diabetic patients receiving islet allografts with Reparixin or placebo.

	Reparixin Median (Range)	Placebo Median (Range)	*p* Value
**Age at 1st ITx (years)**	46 (28–56)	40 (30–48)	*p* = 0.5131
**Sex M/F**	4M/4F	1M/3F	
**Weight at 1st ITx (kg)**	77 (42.6–93.8)	74 (58.0–82.4)	*p* = 0.5131
**BMI at 1st ITx (kg/m^2^)**	26 (18.9–29.9)	25 (21.8–29.9)	*p* = 0.9960
**Pre-ITx A1c (%)**	7.4 (6.8–8.1)	7.0 (6.5–8.6)	*p* = 0.1899
**Pre-ITx Insulin (mg/dL)**	34 (29–67)	36 (29–48)	*p* = 0.8364
**Duration of T1D (years)**	32 (16–44)	31 (15–47)	*p* = 0.9960

ITx, islet transplantation; M, male; F, female; BMI, body mass index; T1D, type-1 diabetes.

## Supplementary Materials

The following are available online at https://www.mdpi.com/article/10.3390/jcm10122708/s1. Supplementary document: Description of transplantation procedures.
