# Supplementary material for: Evaluating the Prognostic Value of Islet Autoantibody Monitoring in Islet Transplant Recipients with Long-Standing Type 1 Diabetes Mellitus
Source: J Clin Med. 2021 Jun 19;10(12):2708. doi: 10.3390/jcm10122708 (PMC8233942; doi:10.3390/jcm10122708)
Supplement: Supplementary file 1 [file jcm-10-02708-s001.zip › jcm-1223473-supplementary.pdf]

Supplementary Materials

eDocument 1. Description of transplantation procedures……………………..……..….........2

This supplementary material has been provided by the authors to give readers additional information about the work.

eDocument 1. Description of transplantation procedures

Our cohort was composed of 8 patients assigned to the Reparixin group and 4 in the placebo group. Pancreata qualified for islet isolation only from donors with a NAIDS above 50 and with a cold ischemia time of less than 12 hours. Islet isolation was performed according to the Collaborative Islet Transplantation (CIT) Consortium protocol utilizing Liberase (Roche, Indianapolis, IN) for enzymatic digestion. The islet isolation product was preserved in an incubator at a temperature of 22 °C for up to 72 hours, including up to 6 hours of storage in the infusion bag prior to transplantation. Ultrasound-guided percutaneous transhepatic portal venous access was obtained by an interventional radiologist under local anesthesia and intravenous moderate sedation. A 4 Fr or 5 Fr end-hole catheter (Kumpe, Cook Medical, Bloomington, IN) was positioned in the main portal vein using fluoroscopy. Islets suspended in Transplant Media (CMRL 1066 Transplant Media) with 1 M HEPES (Corning Cell Sciences, Tewksbury, MA) supplemented 10% human serum albumin were infused under gravity. Portal pressure was measured before, midway, and after islet infusion. For hemostasis, the catheter was retracted into the hepatic parenchymal track, which was then embolized with gelatin sponge (Surgifoam, Ethicon, Somerville, NJ) and detachable coils (Interlock, Boston Scientific, Marlborough, MA). For thromboembolic prophylaxis, heparin at a dose of 70 U/kg of recipient body weight was added to the islet infusion in the bag(s), followed by an intravenous drip for 48 hours titrated to a goal PTT of 50–60 seconds, and then subcutaneous fractionated heparin for 2 weeks.

For induction of immunosuppression, anti-thymocyte globulin (ATG) (Thymoglobulin, Genzyme, Cambridge, MA) was infused in divided doses for a total of 6 mg/kg of patient body weight, starting one day before islet transplantation. One dose of methylprednisolone (1 mg/kg) was given as premedication during the first ATG infusion only. No other steroids were administered in order to avoid confounding the interpretation of the anti-inflammatory effects of Reparixin. Basiliximab (Simulect, Novartis Pharmaceuticals, East Hanover, NJ) at a standard dose of 20 mg was infused intravenously immediately before and 3 days after a second islet transplant. Maintenance immunosuppression included oral mycophenolate mofetil at the target dose of 1000 mg twice a day or mycophenolic acid 720 mg twice a day. The goal for tacrolimus serum level was in the range of 8–10 ng/mL during first 3 months and 7–9 ng/mL afterwards. The dose was adjusted, if medically necessary, when adverse events occurred.

Study procedures performed according to the common multicenter trial protocol. Based on the randomization number, patients received Reparixin at a dose of 2.772 mg/kg/hour or placebo in continuous infusion through a central line over 7 days starting 12 hours before islet transplantation. If insulin independence was not achieved by day 75, each subject received a second islet transplant with repeat infusion of the previously assigned Reparixin or placebo, as performed during the first islet transplant. Islet product release criteria included: viability over 70%, purity over 20%, pellet volume less than 10 mL, islet mass transplanted above 3,000 islet equivalent (IEQ) per recipient body weight in kilograms (IEQ/kg) per transplant.

References:

Witkowski P, Wijkstrom M, Bachul P, Morgan KA, Levy M, Onaca N, Chaidarun SS, Gardner T, Shapiro AMJ, Posselt A, Ahmad SA, Daffonchio L, Ruffini PA, Bellin MD. Targeting CXCR1/2 in the first multicenter, double-blinded, randomized trial in autologous islet transplant recipients. Am J Transplant. 2021 May 25. doi: 10.1111/ajt.16695. Epub ahead of print.

Bachul PJ, Golab K, Basto L, Zangan S, Pyda JS, Perez-Gutierrez A, Borek P, Wang LJ, Tibudan M, Tran DK, Anteby R, Generette GS, Chrzanowski J, Fendler W, Perea L, Jayant K, Lucander A, Thomas C, Philipson L, Millis JM, Fung J, Witkowski P. Post-Hoc Analysis of a Randomized, Double Blind, Prospective Study at the University of Chicago: Additional Standardizations of Trial Protocol are Needed to Evaluate the Effect of a CXCR1/2 Inhibitor in Islet Allotransplantation. Cell Transplant. 2021 Jan-Dec; 30:9636897211001774.
